# Ethnomedicinal validation of *Telfairia occidentalis* L. leaf: a dual experimental and computational approach to uterine leiomyoma therapy

**DOI:** 10.3389/fnut.2025.1669528

**Published:** 2025-12-12

**Authors:** Akingbolabo Daniel Ogunlakin, Gideon Ampoma Gyebi, Amel Elbasyouni, Oyindamola Esther Awosola, Moyosoluwa Mary Dada, Opeyemi Josphine Akinmurele, Abdullahi Adeyemi Adegoke, Israel Kunle Oladoja, Ajibola David Adelakun, Omolola Oluwadara, Gabriel Olalekan Banwo, Adedayo Johnson Adediran, Seun Elizabeth Kuyoro, Adewale Victor Aderemi, Oluyomi Stephen Adeyemi

**Affiliations:** 1 Phytomedicine and Drug Discovery Research Laboratory (PDD-RL), Biochemistry Programme, Bowen University, Iwo, Nigeria; 2 Department of Biotechnology and Food Science, Faculty of Applied Sciences, Durban University of Technology, Durban, South Africa; 3 Department of Molecular Biology and Biotechnology, Pan African University for Basic Sciences, Technology and Innovation (PAUSTI), Nairobi, Kenya; 4 Next Era Pharmacy, Lagos, Nigeria; 5 Department of Physiology, Babcock University, Ilisan Remo, Nigeria; 6 Department of Pharmacognosy, Lead City University, Ibadan, Nigeria; 7 Department of Pharmacognosy, University of Ibadan, Ibadan, Nigeria; 8 Biochemistry Department, Ibrahim Badamosi Babangida University, Lapai, Nigeria; 9 Department of Pharmaceutical Sciences, University of Arizona, Tucson, AZ, United States; 10 Project Development and Design Department, Federal Institute of Industrial Research, Lagos, Lagos State, Nigeria; 11 Department of Veterinary Medicine, University of Ibadan, Ibadan, Nigeria; 12 Directorate of Student Support Services, Bowen University, Iwo, Nigeria; 13 Biochemistry Department, University of Massachusetts, Lowell, Lowell, MA, United States; 14 Department of Medical Biochemistry, Osun State University, Osogbo, Nigeria

**Keywords:** antifibrotic activity, *Telfairia occidentalis*, enzyme inhibition, hormone modulation, computational study

## Abstract

**Introduction:**

A nutrient-dense vegetable with Ethnomedicinal use for treating oxidative and fibrotic diseases is *Telfairia occidentalis* L., often known as “ugu” in Nigeria. Although it has been used extensively in the past, neither experimental nor computational methods have been used to characterize its antifibrotic potential. This study investigates the antioxidant, enzyme-inhibitory, and antifibrotic effects of aqueous *T. occidentalis* leaf extract in albino rats with MSG-induced uterine leiomyomas, employing *in silico modeling* to understand the underlying molecular mechanisms.

**Methods:**

Iron chelation, NO scavenging, and DPPH radical scavenging properties of *T. occidentalis* aqueous extract were evaluated, using quercetin serving as the standard. The inhibitory effects of the extract on α-amylase, α-glucosidase, monoamine oxidase (MAO), and acetylcholinesterase (AChE) were evaluated. Testosterone, FSH, LH, and oestradiol levels were measured in MSG-induced fibroid rats treated with *T. occidentalis* aqueous extract. The tissues of the uterus and ovaries of treated rats were examined histologically. Furthermore, the HPLC-identified compounds in the extract were docked against STEAP4.

**Results and discussion:**

The extract demonstrated modest antioxidant activity; however, it was less effective than quercetin at scavenging NO radicals, DPPH, and iron-chelating capacity. It demonstrated AChE and MAO inhibition that was dose-dependent, with an IC50 for MAO inhibition of 0.178 ± 0.003 μg/mL that was comparable to donepezil (0.155 ± 0.005 μg/mL). α-Amylase activity increased in a dose-dependent manner, whereas α-glucosidase inhibition remained lower than the control. Testosterone and oestradiol levels in *T. occidentalis*-treated fibrotic rats significantly decreased, suggesting that MSG-induced hormonal abnormalities were corrected. Despite some epithelial deterioration, histopathological results showed partial recovery of uterine integrity and restoration of ovarian architecture with growing follicles. These results may suggest that the leaf extract of *T. occidentalis* exhibits antifibrotic and Hormone-modulating properties. HPLC identified beta-carotene and lutein affinity for STEAP4 was discovered by computational methods, suggesting a synergistic process. Through a combination of hormone-regulating, enzyme-inhibitory, and antioxidant properties, *T. occidentalis* shows encouraging antifibrotic efficacy. These results support its traditional application and demonstrate its applicability in the development of phytotherapeutics for the treatment of uterine leiomyomas.

## Introduction

Non-cancerous tumors called uterine leiomyomas, or leiomyomas, grow from the uterus's smooth muscle tissue (1). Infertility, pelvic pain, heavy monthly flow, and frequent urination are some of the most typical symptoms of this gynecological disease that afflict women of reproductive age (2). Some women might not exhibit any symptoms, but others suffer from severe discomfort and consequences that require medical or surgical treatment (3). According to recent research, fibroids cause infertility and hormone imbalance, which in turn increases anxiety and sadness in women of reproductive age (4). Obesity and insulin resistance—both of which are prevalent in diabetes—may encourage the formation of fibroid tumors in women of reproductive age by increasing estrogen and inflammation (5). By the age of 50, up to 70–80% of women worldwide suffer from fibroids, with women of African heritage having the highest frequency (6). Over the previous thirty years, uterine leiomyomas have become more common, especially in low- and middle-income nations. The effect is particularly noticeable in Nigeria (7). According to recent studies, 20–30% of Nigerian women suffer from fibroids; in tertiary hospitals, some regional studies have reported prevalence rates as high as 27.8% in Nigeria (8–10). Many diagnoses occur in women between the ages of 26 and 45, and the condition is a major contributor to gynecological procedures nationwide (11). Although uterine leiomyomas are benign tumors of the uterus's smooth muscles that are not always malignant (12), recent research, however, points to a complicated connection between fibroids and specific cancer types (13). Differentiating between benign and malignant uterine tumors is still difficult to diagnose, even though fibroids seldom develop into leiomyosarcoma (14). Fibroids and estrogen receptor-positive breast cancer have recently been found to be associated, especially in black women (15). These results highlight the necessity of close observation and additional investigation into the common hormonal and genetic pathways that connect cancer and fibroids.

One major financial obstacle in Nigeria is the expense of treating fibroids (2). Non-invasive alternatives such as Uterine leiomyoma Embolisation (UFE) and High-Intensity Focused Ultrasound (HIFU) are even more costly (16, 17). The financial burden is further increased by post-operative care, prescription drugs, and diagnostic testing (18). Many Nigerian women put off or avoid treatment due to high out-of-pocket costs and limited health insurance coverage, which increases health risks and lowers quality of life (19). Early diagnosis, greater knowledge, and better access to reasonably priced care are all necessary to address this expanding public health issue (20). In Nigeria, medicinal plants—including widely consumed vegetables—are increasingly being acknowledged as useful substitutes or supplemental treatments for the treatment of uterine leiomyomas (2). When compared to more traditional therapies like hormone therapy or surgery, these natural cures are prized for their accessibility, cost, and low side effects (21). Numerous botanicals have been shown in recent research to have anti-inflammatory, antioxidant, and hormone-modulating qualities (2, 19). They have also been shown to suppress the growth of fibroid tumors by causing apoptosis and stopping the cell cycle (22). Additionally, vegetables aid in the regulation of estrogen levels, which are crucial for the development of fibroids (23). Herbs and vegetables have long been used in Nigeria to detoxify the liver and lessen estrogen dominance, which shrinks fibroids and eases symptoms like pelvic pain and heavy bleeding (2, 24, 25). For women looking for non-invasive therapies, these botanicals provide a safe and culturally acceptable alternative. The use of medicinal plants in fibroid management has great promise, particularly in resource-constrained locations like Nigeria, where access to advanced care is frequently limited, even though further clinical trials are required to standardize dosages and prove long-term efficacy (26).

The nutrient-rich leafy vegetable, *Telfairia occidentalis* L., often referred to as ugu (Igbo), ewuroko (Yoruba), ikong-ubong (Efik/Ibibio), and okwukwo-wiri (Ikwerre) in Nigeria, is utilized extensively in Nigerian ethnomedicine (27, 28). Its traditional usage in the treatment of infections, inflammation, and anemia has been confirmed by ethnopharmacological research (29). The plant's therapeutic benefits are facilitated by the abundance of bioactive substances, including polyunsaturated fatty acids, alkaloids, flavonoids, saponins, and terpenoids (30). Recent studies have demonstrated the stem extract's analgesic and anti-inflammatory qualities by significantly reducing pain in rat models using tests that cause writhing in response to acetic acid and paw licking in response to formalin (31). Aqueous leaf extracts have also demonstrated neuroprotective and antioxidant properties, lowering oxidative stress indicators and increasing the activity of antioxidant enzymes to lessen the neurotoxicity caused by cyclophosphamide (32). Key contributors to these benefits, especially in regulating inflammation and oxidative damage, have been found as isolated molecules, including 9-octadecenoic acid and linoleic acid methyl ester (33). Additionally, research has documented nephroprotective effects in models of anemia generated by phenylhydrazine (34), indicating promise for the management of renal problems. These highlight *T. occidentalis*' pharmacological significance and encourage its incorporation into evidence-based herbal treatments, particularly in Nigeria. Hence, this study aims to evaluate the antifibrotic effects of *Telfairia occidentalis* leaf aqueous extract using both *in vivo* and *in silico* approaches.

## Materials and methods

### Plant collection and extraction

*Telfairia occidentalis* leaves, devoid of dirt, were collected from Oluponna, Osun State, Nigeria. Identification and authentication were done by Dr. Idowu Obisesan at Bowen University Herbarium, Iwo, which provided the plant with its voucher number (BUH056). For 72 h at room temperature and with sporadic stirring, freshly pulverized leaves were extracted with distilled water. After filtering the extract, the filtrate was concentrated using a freeze-drier. The dark green extract was kept in the refrigerator until further use.

### *In vitro* antioxidant assays

#### 2 ,2-Diphenyl-1-picrylhydrazyl (DPPH) scavenging ability

With minor adjustments, the procedure outlined by Fadogba et al. (35) was utilized to conduct the DPPH radical scavenging assay using 2,2-diphenyl-1-picryl-hydrazyl. Labels were applied to four test tubes: 10 mg/mL, 20 mg/mL, 30 mg/mL, and 40 mg/mL. One milliliter of DPPH solution was added to each of the other test tubes, but two milliliters were added to the control test tube. Each test tube received 1 mL of the test samples; the control tube received nothing. The absorbance of each tube was measured at 516 nm after it had been kept in a pitch-black chamber for 30 min. This procedure was repeated three times.

#### Ferric reducing antioxidant power (FRAP) potential

An adjustment to the procedure outlined by Ogunlakin et al. (36) was used to ascertain the reducing power of the extract. The following four test tubes were labeled thus: control (10 mg/mL), 20 mg/mL, 30 mg/mL, and 40 mg/mL. A volume of 200 μL was transferred to each test tube, followed by the addition of 2 mL of pH 6.6 phosphate buffer and 2 mL of 1% KFeCN. The test tubes were then submerged in a water bath and allowed to incubate at 50 °C for 10 min. Following the incubation period, two milliliters of 10% TCA were added to each test tube. The mixture was then allowed to stand for 15 min, and two milliliters of the supernatant were moved to another test tube. Each supernatant received 500 μL of 0.1% added to it, and a spectrophotometer was used to measure the solution's absorbance at 700 nm.

#### Fenton's reaction (OH Radical scavenging activity)

The ability of the phenolics to prevent Fe^2+^/H_2_O_2_-induced decomposition of deoxyribose was carried out using the method of Olanrewaju et al. (37). Briefly, an appropriate test sample was added to a reaction mixture containing 120 μL 20 mM deoxyribose, 400 μL 0.1M phosphate buffer, 40 μL 20 mM hydrogen peroxide, and 40 μL 500 μM FeSO4, and the volume was made up to 800 μL with distilled water. The reaction mixture was incubated at 37 °C for 30 min, and the reaction was then stopped by the addition of 0.5 mL of 2.8% trichloroacetic acid (TCA); this was followed by the addition of 0.4 mL of 0.6% thiobarbituric acid (TBA) solution. The tubes were subsequently incubated in boiling water for 20 min. The absorbance was measured at 532 nm in a spectrophotometer. The percentage (%) **?**OH radical scavenging ability was subsequently calculated.

#### Determination of Fe^2+^ chelating ability

Twenty microliters of 2 milligrams of FeCl_2_ were mixed with 100 microliters of the extract to make 3 milliliters of ethanol. For 10 min, the mixture was allowed to sit at room temperature after 40 μL of 5 mM ferrozine was added to start the reaction. The sample's absorbance was measured at 562 nm (36).

#### Nitric oxide scavenging activity

The concept underlying the assay is that NO and/or its oxidative derivatives react with a non-fluorescent chemical to produce a fluorescent result (36). The extract and ascorbic acid at different concentrations were combined with 250 μL of 10 mM sodium nitroprusside. After the mixture had been incubated for 180 min at 25 °C, 500 μL of Griess reagent was added, and the absorbance at 546 nm was measured. Control samples contain 250 μL of sodium nitroprusside, phosphate-buffered saline, and Griess reagent but no extract or standard.

#### Inhibition of lipid peroxidation

The female albino rats were anesthetized by intraperitoneal administration of sodium pentobarbital (40 mg/kg), and the cerebral tissue (whole brain) and pancreas were rapidly dissected and placed on ice and weighed. This tissue was subsequently homogenized in cold saline (1:10, w/v) with approximately 10 up-and-down strokes at 1,200 rpm in a Teflon-glass homogenizer. The homogenate was centrifuged for 10 min at 3,000 × g to yield a pellet that was discarded, and a low-speed supernatant (S1) was kept for lipid peroxidation assay. The lipid peroxidation assay was carried out using the modified method of Ogunlakin et al. (36), briefly 100 L S1 fraction was mixed with a reaction mixture containing 30 L of 0.1 M Tris–HCl buffer (pH 7.4), ginger extract (0–100 L) and 30 L of the pro-oxidant solution (7 mM sodium nitroprusside and 15 mM Quinolinic acid). The volume was made up to 300 L with water before incubation at 37 °C for 1 h. The color reaction was developed by adding 300 mL, 8.1% sodium dodecyl sulfate (SDS) to the reaction mixture containing S1; this was subsequently followed by the addition of 500 mL of acetic acid/HCl (pH 3.4) and 500 mL, 0.8% TBA (thiobarbituric acid). This mixture was incubated at 100 °C for 1 h. TBARS (thiobarbituric acid reactive species) produced were measured at 532 nm, and the absorbance was compared with that of a standard curve using malondialdehyde (MDA).

#### Acetylcholinesterase (ACHE) Inhibitory assay

Acetylcholinesterase activity was assessed using the colorimetric method (38). The reaction assay mixture consisted 2,000 mL 100 mM phosphate butter pH 8.0, 100 mL of test sample stock solution in methanol (a final concentration of 42.5 μg/mL), 100 mL, of enzyme AchE solution at a final concentration of 0.03 U/mL and 0.01 μ/mL respectively, 100 μL of DTNB (0.3 mM) prepared in 100 M phosphate buffer pH 7.0 containing 120 mM sodium bicarbonate. The reaction mixture was vortexed and then pre-incubated in a water bath at 37 °C for 30 min. The reaction was then initiated by the addition of 100 μL of ATCI or BTCI at a final concentration of 0.5 mM as a negative control. The inhibitor solution was replaced with methanol. The change in absorbance at λ_max_ 412 nm was then measured for 5 min at ambient temperature. All assays were carried out in triplicate. The final concentration of the sample was 42.5 μg/mL. Donepezil was used as a positive control at the same concentration. The % inhibition was calculated.

#### Monoamine oxidase inhibitory assay

The effect of the extract on MAO (EC 1.4.3.4) activity was measured according to a previously reported method (39). In brief, the reaction mixture contained 0.025 M phosphate buffer (pH 7.0), 0.0125 M semicarbazide, 10 mM benzylamine, 0.67 mg of the enzyme, and 0–100 μL of extract. After 30 min incubation, acetic acid was added and boiled for 3 min in a boiling water bath, followed by centrifugation. The resulting supernatant (1 mL) was mixed with an equal volume of 2, 4-dinitrophenylhydrazine, and 1.25 mL of benzene was added after 10 min of incubation at room temperature. After separating the benzene layer, it was mixed with an equal volume of 0.1 N NaOH. The alkaline layer was decanted and heated at 80 °C for 10 min. The orange–yellow color developed was measured at 450 nm in a UV/visible spectrophotometer (Jenway 6305 model). The MAO activity was thereafter expressed as a percentage inhibition of the reference.

### *In vitro* antidiabetic assay

#### α-amylase inhibitory potential

This assay was conducted following the standard protocol to determine the α-amylase inhibitory potential of the extracts (37). To begin, fresh preparation of the enzyme was prepared, comprising 5 units per milliliter, in pH 6.7 ice-cold PBS with a concentration of 20 mM and 6.7 mM NaCl. Then, 250 μL of the enzyme was combined with inhibitors (acarbose or test samples) at varying concentrations (excluding a blank sample), and the mixture was incubated at 37 °C for 20 min. Then, a starch solution at a concentration of 0.5% (w/v) was added, and the mixture was incubated for an additional 15 min at 37 °C. Immediately after the DNS reagent was added, the mixture was mixed and placed in a water bath at 100 °C for 10 min. Finally, the absorbance was read at 540 nm.

#### α-glucosidase inhibitory potential

The effect of the extracts on intestinal α-glucosidase activity was evaluated using a technique described by Mechchate et al. (40), which quantified the glucose generated by sucrose breakdown. To perform the assay, 100 μL of sucrose (50 mM), 1,000 μL of phosphate buffer (50 mM; pH = 7.5), and 100 μL of α-glycosidase enzyme solution were prepared as the test solution (10 I.U.). Control (distilled water), positive control (acarbose), or test samples were all added to this mixture at varying concentrations. The absorbance was read at 500 nm.

### *In vivo* assay

#### Dosing of experimental animals

Human therapeutic dosages of *T. occidentalis* range from 1 to 9 g, according to the ethnobotanical survey that was carried out in Iwo, Nigeria, during the study (unpublished data). Rat dosage was determined based on body surface area and the conversion factor from human to albino rat (conversion factor = 0.162). To do this, it was divided by the 60 kg adult human weight and then multiplied by a factor to account for the animal's body surface area using the procedure of Nair and Jacob (41), which was further explained by Ogunlakin et al. (42, 43) and Ige et al. (19). About 100 mg/kg bw is the range of the calculated dose that was attained. Thus, in this investigation, a dose of 100 mg/kg was used. Gavage was used to dose albino rats. During the experiment, the dosage was once daily at a volume of 2 milliliters per kg body weight. Based on the animal's most recent reported body weight, individual dose volumes were computed. Since oral delivery is the recommended method of human exposure, it was chosen.

#### Ethical approval and experimental animals

Healthy female albino rats (15) weighing 190 and 200 g (8 weeks old) were procured from the Biochemistry programme, Bowen University in Iwo, Nigeria. All experimental rats used in this study were handled in accordance with the rules and regulations established for animal management in research, as outlined in NIH Publications No. 80-23 revised, 1996. Authorization number BUI/BCH/2025/001 was issued for the study, and the rats were kept following the guidelines set forth by the Institutional Animal Ethics Committee of the Department of Biochemistry at Bowen University, Iwo (BUI). The albino rats were handled with care and housed in plastic cages within a clean, well-ventilated animal facility at Bowen University, Iwo. Environmental conditions, including temperature and humidity, were appropriately maintained. The rats received standard pellet feed and had unrestricted access to water. A natural 12-h light/dark cycle was observed, and all animals were acclimatized for 1 week before experimentation.

#### MSG-induced uterine leiomyoma study

Female albino rats were grouped into three groups of five animals each. The experimental study was organized into three distinct groups to evaluate the effects of *T. occidentalis* aqueous extract on monosodium glutamate (MSG) induced-uterine leiomyoma. Group A, serving as the control group, did not receive any MSG (Vedan Limited) or *T. occidentalis* aqueous extract. This group remained untreated throughout the 30 days. Group B was administered MSG at a dosage of 800 mg/kg daily for 30 days to induce uterine leiomyoma. However, Groups A and B received 1.2 mL of distilled water for 30 days. Group C underwent the same induction protocol as Group B, receiving 800 mg/kg of MSG for 30 days to induce uterine leiomyoma. Following this induction phase, Group C was treated with 100 mg/kg of *T. occidentalis* aqueous extract daily for an additional 30 days. This design allowed for comparative analysis between untreated, induced-only, and induced-plus-treated groups. After 24 h of the last treatment, the rats were anesthetized by intraperitoneal administration of sodium pentobarbital (40 mg/kg) at the end of the experiment.

### Hormonal analysis

#### Total serum follicle-stimulating hormone (FSH)

Follicle-stimulating hormone (FSH) levels were quantified using a direct sandwich enzyme-linked immunosorbent assay (ELISA), following the protocol provided by Calbiotech Inc. (FS046F). In this assay, each sample was combined with an anti-FSH-HRP conjugate and added to wells pre-coated with monoclonal anti-FSH antibodies. After incubation, any unbound proteins were removed through a washing step. The substrate was then introduced, initiating a colorimetric reaction. The resulting color intensity was measured spectrophotometrically at a wavelength of 450 nm.

#### Luteinizing hormone (LH)

The serum levels of luteinizing hormone (LH) were measured using a solid-phase enzyme-linked immunosorbent assay (ELISA), specifically the Calbiotech Inc. kit (LH231F). This assay employs a sandwich enzyme immunoassay technique, wherein LH present in the sample binds to microwells that have been pre-coated with streptavidin. A conjugate reagent is then introduced, followed by the addition of the substrate tetramethylbenzidine (TMB). After a period of incubation and subsequent washing to remove unbound components, the resulting color intensity is quantified using a spectrophotometer set to 450 nm.

#### Estradiol level

Serum estradiol (E2) concentrations were measured using a competitive binding enzyme-linked immunosorbent assay (ELISA), following the manufacturer's protocol provided by Calbiotech Inc. (ES380S). In this assay, each sample was incubated with an anti-E2 antibody and an enzyme-linked estradiol conjugate. The conjugated antigen competes with the endogenous estradiol in the sample for the limited antibody binding sites. After incubation, the reaction was halted using an acid solution, and the absorbance was measured at 450 nm. Notably, the color intensity observed was inversely proportional to the estradiol concentration in the sample.

#### Testosterone level

Testosterone levels were assessed using the DRG EIA-1559 ELISA kit, following the manufacturer's protocol provided by DRG Instruments GmbH, Germany. In brief, 25 μL of blood plasma was dispensed into each well designated for standards, samples, and controls. Subsequently, 200 μL of enzyme conjugate was added to each well and gently mixed for 10 s. The plate was incubated at room temperature for 60 min, after which it was washed three times with 400 μL of wash buffer per well. To ensure complete removal of residual liquid, the plate was firmly blotted on absorbent paper. Next, 200 μL of substrate solution was added to each well and allowed to react for 15 min at room temperature. The enzymatic reaction was then halted by adding 100 μL of stop solution to each well. Absorbance was measured at 450 nm using the xMark™ Microplate Absorbance Spectrophotometer (Bio-Rad Laboratories Inc.). Testosterone concentrations were calculated using Microplate Manager^®^ 6 Software (Bio-Rad Laboratories Inc.).

#### Ovarian and cervical histology

Following an overnight fast, the animals underwent laparotomy to facilitate the collection of ovaries and cervixes. The tissue architecture was examined using the standard histological method described by Avwioro (122), employing the Haematoxylin and Eosin staining technique. The harvested tissues were carefully dissected into small fragments, each no more than 4 mm thick, and placed into pre-labeled cassettes. These fragments were then fixed in 10% formal saline for 24 h. The formal saline solution was prepared by combining 100 mL of 40% formaldehyde, 9 g of sodium chloride (NaCl), and 900 mL of distilled water. Automated tissue processing was carried out using the Leica TP 1020 tissue processor. The samples were dehydrated through a graded series of alcohol concentrations, 70%, 80%, 90%, and 95%, alongside formal saline. Once dehydrated, the tissues were embedded in molten paraffin wax, poured into metal molds, and transferred to a cold plate to solidify. After solidification, the paraffin blocks were removed from the molds and trimmed to expose the tissue surface. Sectioning was performed using a rotary microtome at a thickness of 6 μm. These sections were further sliced into 4 μm ribbon sections and floated on a water bath (Raymond Lamb) maintained at 55 °C. The sections were then mounted onto clean, labeled slides, dried on a hotplate (Raymond Lamb) set at 60 °C for 1 h, and subsequently examined under a light microscope using × 100 and × 400 objective lenses.

#### HPLC analysis

To identify the phytochemical constituents present in the *T. occidentalis* extract, High-Performance Liquid Chromatography (HPLC) analysis was performed following the protocol described by Olanrewaju et al. (37), with slight modifications to optimize separation conditions. The analysis was carried out using a reverse-phase C18 column (250 mm × 4.6 mm, 5 μm particle size), maintained at a constant temperature of 30 °C. The mobile phase consisted of solvent A (water containing 2% acetic acid) and solvent B (methanol), applied in a gradient elution profile: starting with 5% methanol for the first 2 min, followed by incremental increases in methanol concentration every 10 min from 10 to 60 min. The flow rate was set at 1.0 mL/min, and the injection volume for each sample was 20 μL. Detection of eluted compounds was achieved using a UV detector set at a wavelength of 254 nm, which is suitable for monitoring a broad range of phytochemicals. The compounds detected were further identified by comparing their spectral data against entries in the National Institute of Standards and Technology (NIST) library. Specifically, retention times and UV absorption profiles obtained during HPLC runs were matched with known reference spectra in the NIST database to confirm the identity of each phytochemical.

### Molecular docking studies of identified compounds against the target

#### Protein structure preparation

The 3D-structures of STEAP4 bound to NADPH, FAD (PDBID: 6HD1) was retrieved from the Protein Data Bank (http://www.rcsb.org). The existing ligands and water molecules were removed from all the crystal structures, while missing hydrogen atoms were added using MGL-AutoDockTools (ADT, v1.5.6) (44).

#### Ligand preparation

The retrieval of the Structure Data Format (SDF) of *T. occidentalis* identified bioactive compounds were downloaded from the PubChem database (www.pubchem.ncbi.nlm.nih.gov). The compounds were further converted to the PDB chemical format by means of Open Babel (45). Non-polar hydrogen molecules were merged with the carbons, while the polar hydrogen charges of the Gasteiger-type were assigned to atoms. Furthermore, ligand molecules were converted to dockable PDBQT format with the help of AutoDock Tools.

#### Molecular docking of phytochemicals with the targeted active site

AutoDock Vina integrated with PyRx 0.8 was used to carry out an active site target molecular docking of the reference inhibitors, and the LCMS identified bioactive compounds to the binding site of the target protein (46). Before the docking analysis, PyRx 0.8's bioactive chemicals were imported using Open Babel (45). The bioactive compounds were further minimized by Open Babel. The energy minimization parameter and conjugate gradient descent used were the Universal Force Field (UFF) and optimization algorithm, respectively. The binding site coordinates of the target proteins were identified by mapping the amino acid residues around the binding site of the native ligand. The dimension of the grid boxes formed was center *x* (−28.25), *y* (26.12), *z* (−22.72), and size *x* (33.95), *y* (36.43), *z* (54.20). The selected conformer from the docking analysis was further subjected to interactive analysis using Discovery Studio Visualizer version 16.

#### Molecular dynamics

For a 100 ns molecular dynamics simulation, the complexes of the top two docked compounds with the STEAP4 (6HD1) were further chosen. The study was conducted using GROMACS 2019.2 and the GROMOS96 43a1 force field (47–49). The proteins and ligands topology files were generated using the Charmm GUI (50, 51). The simulation used a solvation system, periodic boundary conditions, physiological conditions, system minimization, equilibration in a constant number of atoms, constant pressure, and constant temperature (NPT), all of which were similar to those in our previous study (42, 43, 52). The velocity rescales and Parrinello-Rahman barostat were used to maintain the temperature and pressure at 310 K and 1 atm, respectively. A 2-femtosecond time step was used with a leapfrog integrator. Each system underwent a 100 ns simulation, with snapshots taken every 0.1 ns and totaling 1000 frames for each system. From the MDs trajectories, the RMSD and RMSF, ROG, SASA, and H-bonds.

#### Binding free energy calculation using MM-GBSA

The Molecular Mechanics Generalized Born Surface Area (MM-GBSA) method and decomposition analysis using the gmx MMPBSA package were used to obtain the binding energies of amino acids within 0.5 nm of the ligand in order to determine the binding free energy of the two top docked phytochemicals from the initial docking analysis (53, 54). The methods used were the same as those published in our previous manuscripts (55, 56)

### Statistical analysis

Values were displayed as Mean ± Standard deviation (SD). Data were analyzed using one-way analysis of variance (ANOVA), and group means were compared using Dunnett's Multiple Comparison and Bonferroni tests using GraphPad Prism version 5.01 for Windows, GraphPad Software, San Diego, California, USA. *P*-values that were less than 0.05 were deemed significant.

## Results

### Antioxidant and Inhibition of α-amylase, α-glucosidase, cholinesterase, and monoamine oxidase activities

**Figure 1:**
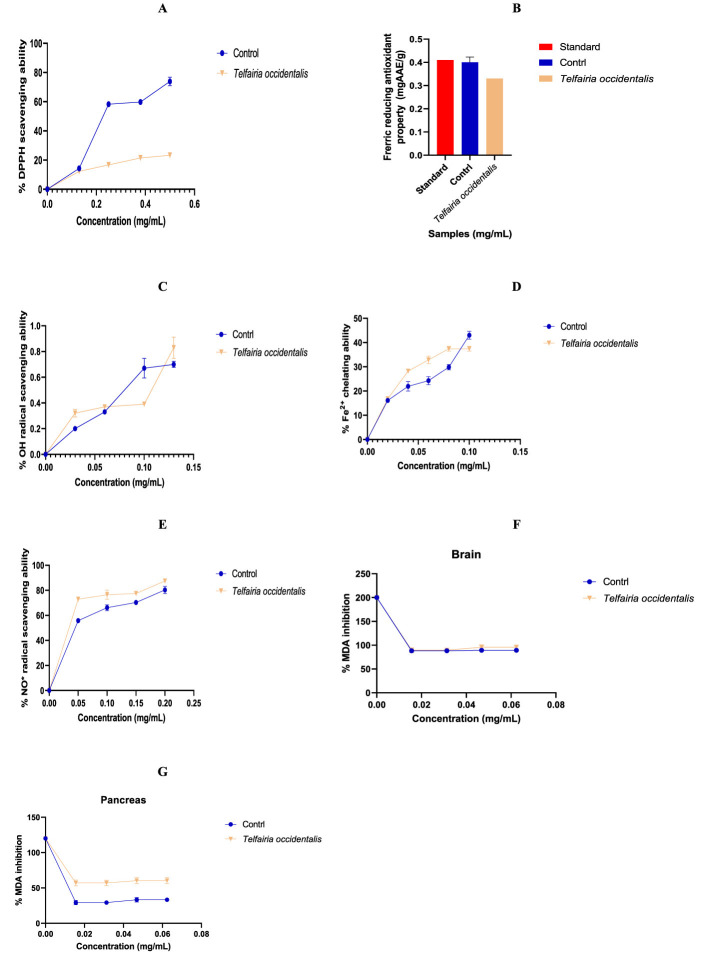
Effects of *T. occidentalis* leaves aqueous extract percentage **(A)** DPPH radical scavenging property, **(B)** Ferric Reducing antioxidant properties, **(C)** Hydroxyl radical scavenging ability, **(D)** Iron Chelating activity, **(E)** Nitric oxide radical scavenging ability, and Malondialdehyde in **(F)** brain and **(G)** pancreas tissues. Control for all is Quercetin, and DPPH radical assay, where ascorbic acid was used. Control for the MDA inhibition assay is Donepezil.

**Figure 2:**
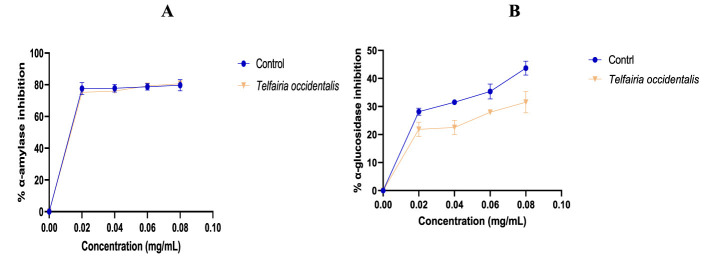
Percentage α-amylase **(A)** and α-glucosidase **(B)** inhibitory effects of *T. occidentalis* leaf. Control (gallic acid).

**Figure 3:**
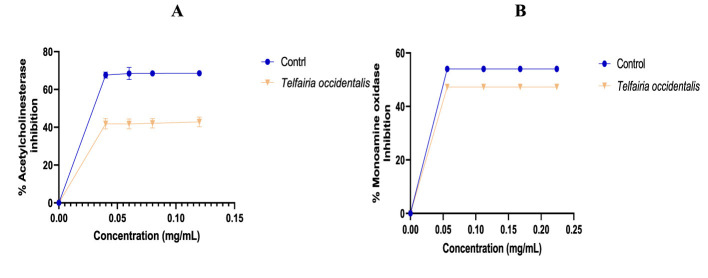
Percentage Acetylcholinesterase (ACHE) **(A)** and Monoamine oxidase **(B)** inhibitory effects of *T. occidentalis* leaf. Control (Donepezil).

The antioxidant activity of the aqueous extract of *T. occidentalis* leaves is displayed in Table 1. When compared to quercetin, the standard utilized, the extract was found to have a lesser capacity to scavenge DPPH radicals. It is demonstrated that the NO radical ability of the extract was lower to quercetin (Figure 1), the criterion that was employed. The extract's capacity to chelate iron (Fe^2+^) was higher than that of the employed control, quercetin. The α-amylase activity of the extract was equivalent to that of the control, as seen in Table 2. As extract concentrations rise, Figure 2A illustrates an increase in α-amylase activity. In Figure 2B, the extract's capacity to inhibit α-glucosidase activity was lower than that of the control. The effects of *T. occidentalis* leaf extract on acetylcholinesterase (AChE) and monoamine oxidase (MAO) activities are presented in Figures 3A, B, respectively. The extract exhibited dose-dependent inhibitory activity against both enzymes. Similarly, *T. occidentalis* extract significantly inhibited MAO activity in a dose-dependent manner, with an IC50 value of 0.176 ± 0.003 μg/mL, which was slightly higher than that of donepezil (0.155 ± 0.005 μg/mL) as shown in Table 3.

**Table 1:** Antioxidant potentials of *T. occidentalis* leaves aqueous extract expressed as IC_50_ values (mg/mL).

**Samples**	**IC**_**50**_ **(mg/mL)**					
	**DPPH scavenging**	**Fe Chelation**	**NO scavenging**	**OH radical scavenging**	**MDA (Brain)**	**MDA (Pancreas)**
*T. occidentalis*	0.926 ± 0.010^*^	0.108 ± 0.010^**^	0.092 ± 0.010^**^	8.812 ± 0.010^**^	0.024 ± 0.002^NS^	0.024 ± 0.005^NS^
Standard(s)	0.311 ± 0.010^Asc^	0.118 ± 0.010^Q^	0.014 ± 0.010^Q^	8.534 ± 0.010^Q^	0.026 ± 0.002^D^	0.026 ± 0.005^D^

Asc = ascorbic acid; Q = quercetin; D = Donepezil; NS means there is no significant difference, and ^*^ and ^**^ mean there is a significant difference compared to the standard.

**Table 2:** α-amylase and α-glucosidase inhibitory activity of *T. occidentalis* leaves aqueous extract expressed as IC_50_ values (mg/mL).

**Samples**	**IC**_**50**_ **(mg/mL)**	
	α**-amylase inhibitory activity**	α**-glucosidase inhibitory activity**
*T. occidentalis*	0.038 ± 0.004^*^	0.062 ± 0.003^**^
Standard	0.038 ± 0.002	0.081 ± 0.005

Standard = Gallic acid.

^*^ and ^**^ means there is a significant difference compared to the standard.

**Table 3:** Acetylcholinesterase and Monoamine oxidase Inhibitory activity of *T. occidentalis* leaves aqueous extract expressed as IC_50_ values (mg/mL).

**Samples**	**IC**_**50**_ **(mg/mL)**	
	**Acetylcholinesterase inhibitory activity**	**Monoamine oxidase inhibitory activity**
*T. occidentalis*	0.089 ± 0.005^**^	0.176 ± 0.003^**^
Standard	0.061 ± 0.009	0.155 ± 0.005

Standard = Donepezil.

^**^means there is a significant difference compared to the standard.

### *In vivo* study

**Figure 4:**
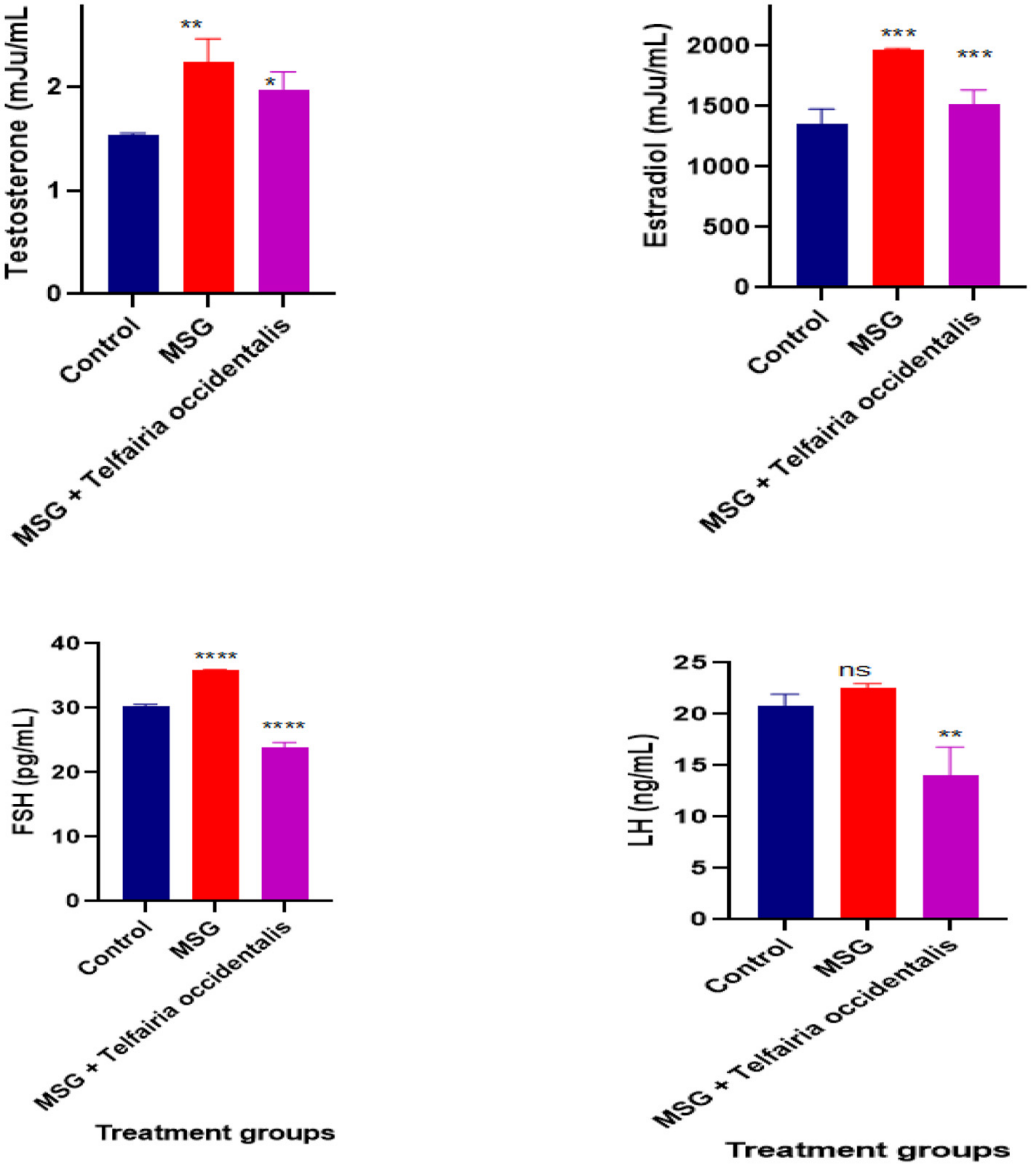
Effect of *T. occidentalis* leaf extract on FSH, LH, Testosterone, and Estradiol levels in control and treated rats. ***, ***, and **** indicate a statistically significant difference compared to the control group.

**Figure 5:**
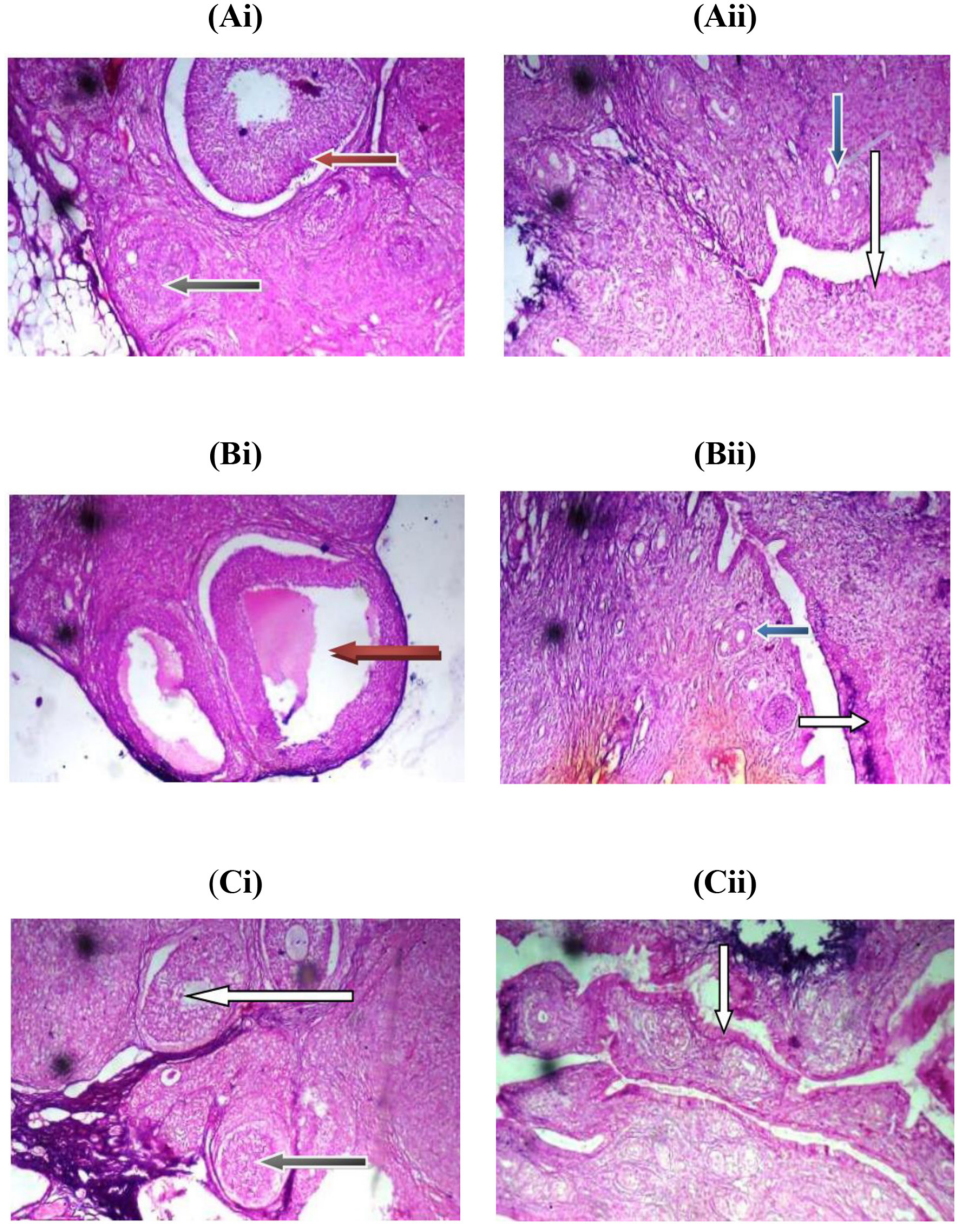
Photomicrograph of ovaries and uterine sections of the control **(Ai** and **Aii)**, MSG-induced **(Bi** and **Bii)**, and *T. occidentalis*-treated **(Ci** and **Cii)** groups, respectively, stained by hematoxylin and eosin (Mag. ×40). For the ovary, the Graafian follicle (red arrow), and Atresia follicle (black arrow) within the ovarian cortex, antral follicle with oocyte (green arrow). For the cervix, the endometrium epithelial layer (white arrow), normal endometrial gland (blue arrow).

While the levels of luteinizing hormone (LH) in the MSG-induced group and the *T. occidentalis*-treated group were not similar, Figure 4 demonstrates that the MSG-induced uterine leiomyoma rats had a significantly higher level of testosterone. *T. occidentalis* successfully corrected the hormonal anomalies in the albino rats, as evidenced by the treatment group's reduced levels of testosterone and oestradiol in comparison to the control group. The photomicrograph of the rats' uterus and ovaries is shown in Figure 5. A photomicrograph of the *T. occidentalis*-treated group's ovary section revealed growing follicles in the ovarian cortex, including atretic, antral, and secondary follicles. The ovarian stroma exhibits mild vascular congestion, luteinized cells, and typical connective tissue. Additionally, an endometrial epithelial layer with deteriorated epithelial cells, displaying vacuolation and necrosis, was visible in photomicrographs of a uterine segment. Proliferative endometrial cells inside a significantly infiltrated stroma and endometrial glands with vacuolated epithelial cells are also visible.

### Phytochemistry

**Figure 6:**
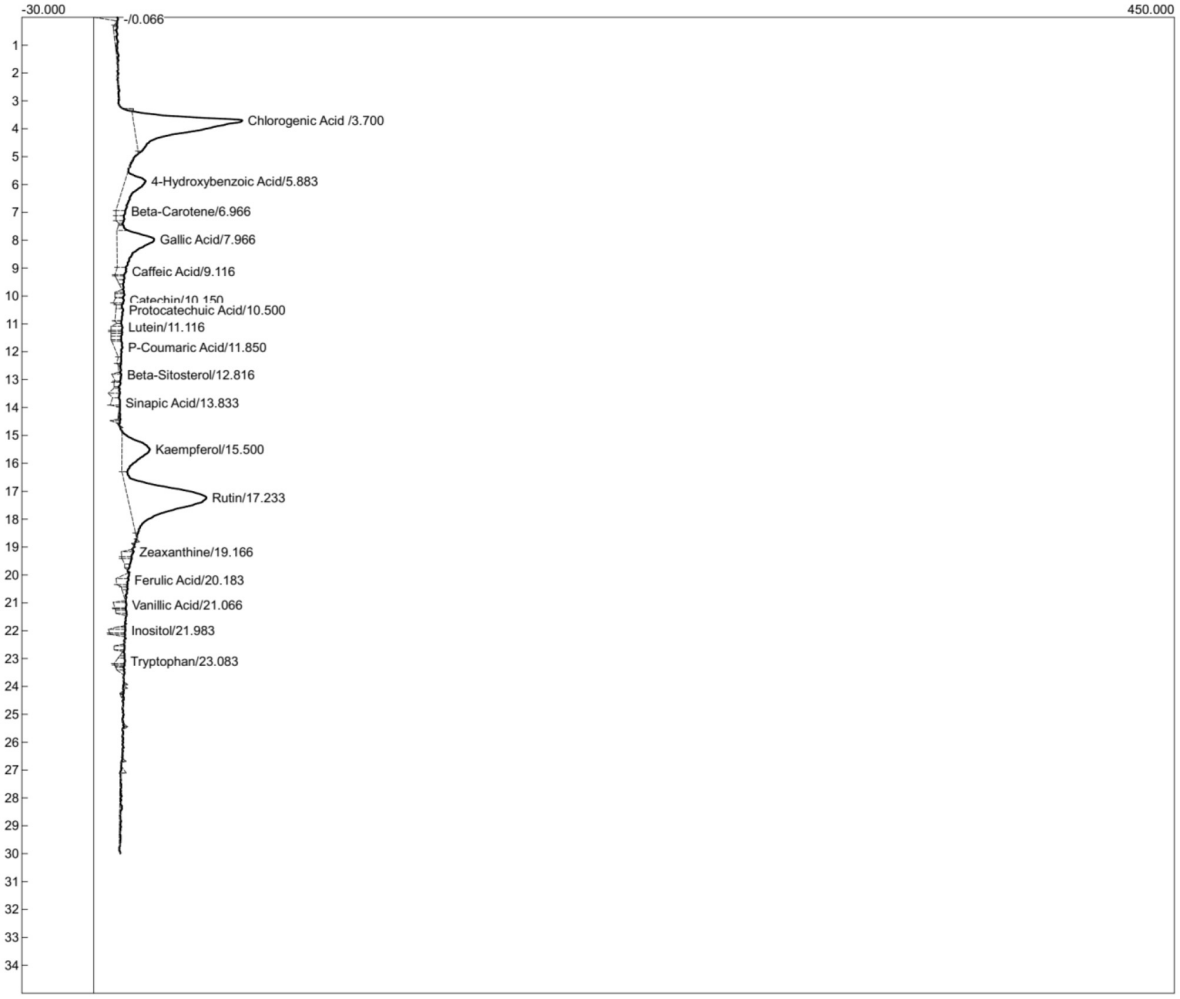
HPLC chromatogram of *T. occidentalis* leaf aqueous extract.

The HPLC chromatogram (Figure 6) revealed the presence of 17 bioactive compounds, with retention times ranging from 3.7 to 23.08 min. These compounds include chlorogenic acid, gallic acid, kaempferol, beta-sitosterol, rutin, beta-carotene, lutein, zeaxanthin, inositol, tryptophan, and ferulic acid.

### *In silico* study

#### Molecular docking

**Figure 7:**
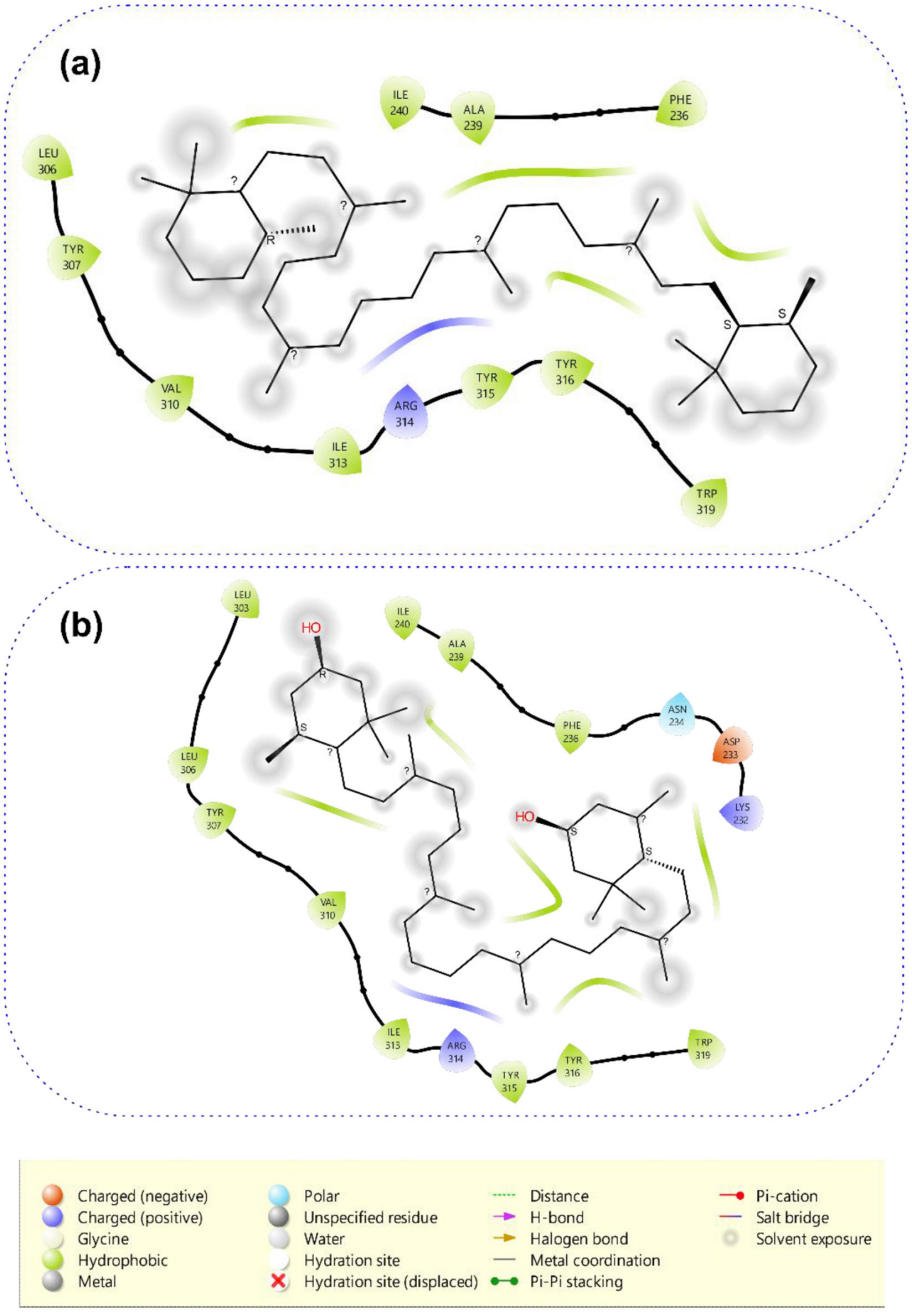
Interactive plots of *Telfairia occidentalis* identified compounds from the docking analysis with amino acids in the binding site of STEAP4. The ligands are displayed as sticks.

Table 4 displays the binding scores of the bioactive chemicals discovered by T. occidentalis against STEAP4. With a docking score of −9.8 kcal/mol, beta-carotene demonstrated the strongest binding affinity, followed closely by lutein and rutin at −9.7 and 9.5 kcal/mol, respectively, according to the docking values, which represent the estimated free binding energy (kcal/mol). With scores of −9.2 kcal/mol, zeaxanthine and kaempferol both showed noteworthy binding potentials. Mostly hydrophobic interactions, such as pi-sigma contacts with Phe236 and Trp319 and pi-alkyl contacts with Trp319, Phe236, and Leu306, stabilized beta-carotene's binding to STEAP4 (6HD1) (Figure 7). Alkyl contacts accounted for the remaining interactions, which included those with Tyr307, Ile240, Ile313, Arg314, Tyr316, Tyr315, Ala239, Val310, and Tyr315. Hydrophobic interactions also stabilized lutein, such as alkyl contacts with Ala239, Leu306, Val310, Phe236, Tyr307, Trp319, and Arg314, pi-sigma contacts with Trp319, and pi-alkyl contacts with Trp319, Phe236, Leu306, and Tyr307.

**Table 4:** Binding scores of *T. occidentalis* HPLC-identified bioactive compounds against STEAP4.

**(S/N)**	**Identified compounds**	**Docking scores (Kcal/mol)**
1	Beta-Carotene	−9.8
2	Lutein	−9.6
3	Rutin	−9.6
4	Kaempferol	−9.2
5	Zeaxanthine	−9.2
6	Beta-Sitosterol	−8.9
7	Chlorogenic Acid	−8.8
8	Catechin	−8.3
9	Caffeic Acid	−6.7
10	Tryptophan	−6.7
11	Ferulic Acid	−6.6
12	Protocatechuic Acid	−6.4
13	P-Coumaric Acid	−6.4
14	Vanillic Acid	−6.4
15	Sinapic Acid	−6.3
16	Gallic Acid	−6.1
17	4-Hydroxybenzoic Acid	−6
18	Inositol	−6

#### Molecular dynamics

**Figure 8:**
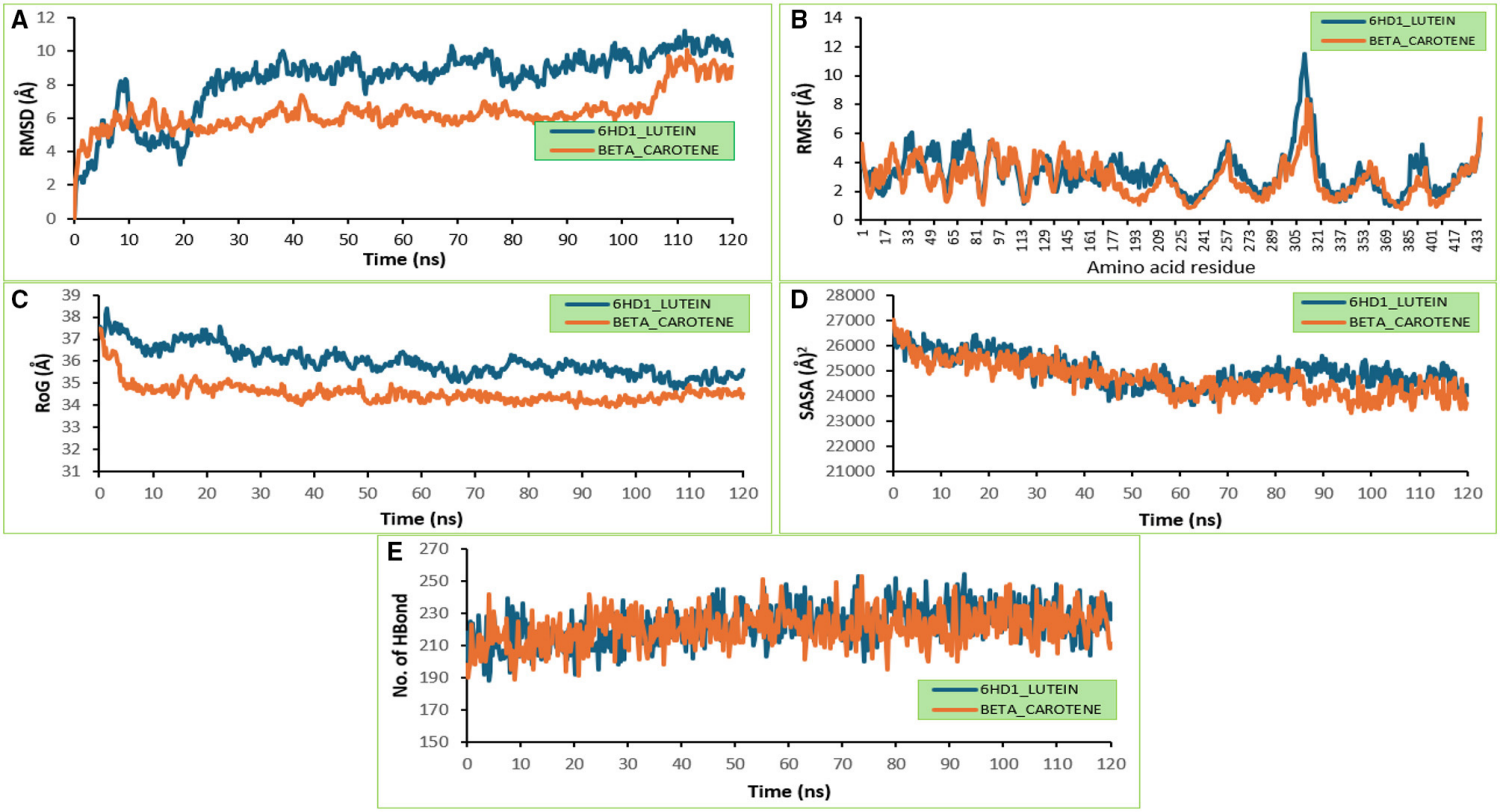
The plots of thermodynamic parameters computed from the analysis of the MDs trajectories of unbound and bound 6HD1 complex systems, **(A)** The Backbone-Root Mean Square Deviation (RMSD), **(B)** Per residue Root Mean Square Fluctuations (RMSF), **(C)** radius of gyration, **(D)** Surface Accessible Surface Area (SASA), **(E)** number of hydrogen atoms.

Following a 10-ns equilibration period, the RSMD systems for both complexes displayed distinct progressions until the simulation's conclusion (Figure 8A). Table 5 displays the means and standard deviations of several characteristics that were obtained from the MDS trajectories of the compounds that were highest-docked and complexed with their respective targets. The greatest variability around amino acid residues between 289 and 337 was shown in the plot of RMSF systems for both complexes (Figure 8B). Figure 8C displays a lower RoG (34.56 Å) for the beta-carotene-bound 6HD1 system and a higher RoG (35.99 Å) for the lutein-bound 6HD1 system. The mean SASA values further supported this (Figure 8D). With a variable fluctuation throughout the simulation period, the plots for the number of hydrogen bonds in both systems were equilibrated at the start of the simulation (Figure 8E). With an average of 223.15 ± 11.3 hydrogen bonds, the 6HD1_lutein complex formed marginally more than beta-carotene (220.76 ± 10.7).

**Table 5:** The means and standard deviations of several parameters derived from the MDS trajectories of the highest-docked compounds complexed with their corresponding targets.

**Interactions**	**RMSD (Å)**	**RMSF (Å)**	**RoG (Å)**	**SASA (Å^2^)**	**H-Bonds**
6HD1_LUTEIN	8.27 ± 1.94	3.29 ± 1.47	35.99 ± 0.65	24975.2 ± 614.9	223.15± 11.3
6HD1_BETA-CAROTENE	6.33 ± 1.12	2.86 ± 1.24	34.56 ± 0.44	24664.4 ± 664.6	220.76 ± 10.7

#### Molecular mechanics generalized born surface area (MMGBSA) analysis

**Figure 9:**
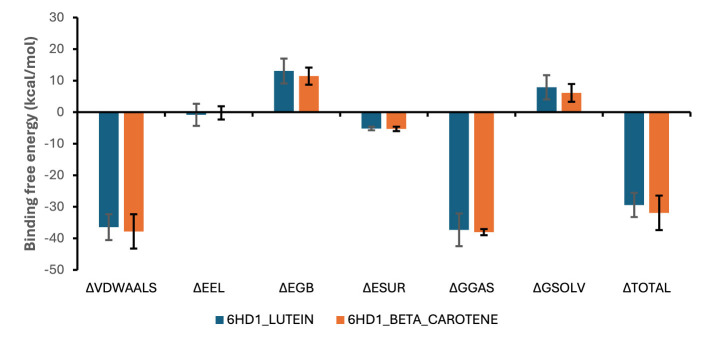
The mean and SD of different energy components that make the binding free energy (kcal/mol) of top docked compounds to 6HD1.

**Figure 10:**
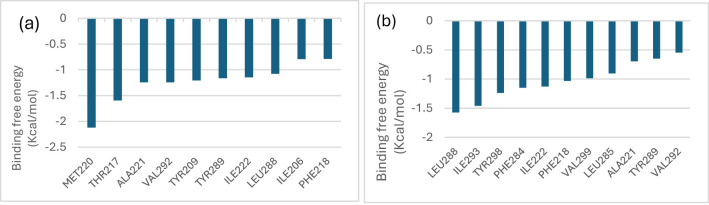
Per-residue decomposition of total binding free energy of the most contributing amino **(a)** 6HD1_lutein, **(b)** beta-carotene systems.

Figure 9 displays the mean and standard deviation of the various energy components that contribute to the binding free energy (kcal/mol) of the top docked molecules to 6HD1. With a total binding energy of −17.07 kcal/mol, lutein provided the most advantageous contribution. Small but beneficial contributions (about −1.07 to −1.20 kcal/mol) were made by residues such as Tyr209, Tyr289, Ile222, and Leu288; these contributions were primarily from hydrophobic interactions (Figure 10a). Strong van der Waals interactions (−18.90 kcal/mol) and moderate polar solvation penalties, along with negligible electrostatic contributions, allowed beta-carotene to contribute the highest favorable binding energy (−18.98 kcal/mol) (Figure 10b).

## Discussion

### *In vitro* antioxidant study

Despite not being the strongest antioxidant in all conventional tests, *Telfairia occidentalis*, or fluted pumpkin, has a strong bioactivity profile that makes it a viable option for phytotherapeutic uses. Its aqueous leaf extract has a moderate ability to scavenge free radicals, but it had considerable metal chelation and lipid peroxidation activities, which are vital in reducing oxidative stress. As reported in literature, Moringa oleifera and curcumin from Curcuma longa outperform *T. occidentalis* in DPPH radical scavenging potential (57–61). Furthermore, there is no direct information on *T. occidentalis*'s neuroprotective and anti-inflammatory properties in neurotoxicity models, nor specific details about its reversal of increased MDA and inflammatory cytokines or increased antioxidant enzyme activity in the literature. Quercetin exhibits a strong iron-chelating capacity, with its effectiveness attributed to its ability to chelate iron and reduce oxidative stress. The aqueous extract of Foeniculum vulgare Mill seeds demonstrated a significant capacity for nitric oxide (NO) scavenging, with an IC_50_ value of 30.91 ± 0.49 mg/mL (Benabdallah et al., 2022). Furthermore, the ethanolic extract of Terminalia bellerica fruit pulp showed strong hydroxyl radical scavenging activity with an IC_50_ of 27 μg/mL.

Lipid peroxidation is a critical indicator of oxidative damage, stemming from reactive oxygen species avidly attacking polyunsaturated fatty acids in cell membranes (62). Various metal ions, particularly iron (Fe^2+^ and Fe^3+^), can activate lipid peroxidation processes in tissues like the adrenal cortex (63, 64). Chelation therapy is an effective strategy for managing heavy metal toxicity and reversing oxidative stress by removing toxic metals and inhibiting lipid peroxidation (65–68). From our study, *T. occidentalis* had a significant reducing effect on malondialdehyde (MDA) concentrations in pancreatic and brain tissues. *Telfairia occidentalis* leaf extract has been observed to reverse oxidative damage, including a significant decrease in malondialdehyde, in albino rats (69). Similarly, the aqueous extract of Terminalia chebula fruit, Terminalia laxiflora methanol extract, Myracrodruon urundeuva (Anacardiaceae), Terminalia brownii methanolic bark extract, and Cassia occidentalis (70) have demonstrated exceptional anti-oxidative properties. This suggests the effectiveness of medicinal plants and vegetables in neutralizing the effects of radicals.

### *In vitro* α-amylase and α-glucosidase inhibitory activities

The ability of *T. occidentalis* to block the α-amylase and α-glucosidase enzymes provides strong support for its possible use in the treatment of type 2 diabetes and postprandial hyperglycemia (71). Strong enzyme inhibition is shown by the IC50 values of 0.038 ± 0.004 mg/mL for α-amylase and 0.062 ± 0.003 mg/mL for α-glucosidase, which is supported by Oluwagunwa et al. (72). The inhibition of α-amylase matches the standard (0.038 ± 0.002 mg/mL), but that of α-glucosidase exceeds it (standard: 0.081 ± 0.005 mg/mL. According to these findings, *T. occidentalis* might provide a dual inhibitory mechanism that has potential use in medicine (71, 72). While inhibiting α-amylase reduces the breakdown of starch into glucose, inhibiting α-glucosidase delays the intestinal absorption of glucose (71). These systems work together to control blood glucose rises that occur after meals (71, 72). Commonly used pharmaceutical inhibitors like acarbose and miglitol frequently result in gastrointestinal adverse effects like flatulence and bloating (73). Because of this, there is now more interest in plant-based substitutes that have fewer negative consequences (71). The antidiabetic qualities of Moringa oleifera are the subject of much research. Aqueous leaf extracts were used by Jimoh (74) to report IC50 values of 0.045 mg/mL for α-amylase and 0.063 mg/mL for α-glucosidase (74). Particularly in terms of α-glucosidase inhibition, these figures are similar to those of *T. occidentalis* (72, 74). A more balanced dual-enzyme inhibitory profile is suggested by *T. occidentalis*' somewhat stronger α-glucosidase inhibition. Furthermore, *T. occidentalis* remains effective against both enzymes. On the other hand, Nigella sativa extract inhibits both α-amylase and α-glucosidase; however, with higher efficacy against α-glucosidase than α-amylase (75). *T. occidentalis*'s diverse phytochemical profile, which includes flavonoids, tannins, and phenolic acids, most likely mediates the enzyme inhibition it exhibits (33, 76). Blanching lowers its enzyme inhibition, indicating that fresh leaves retain more bioactive compounds (77). Furthermore, the mechanism of action of *T. occidentalis* has been supported by docking experiments that have demonstrated significant binding affinities between its phytochemicals and the active sites of enzymes. *T. occidentalis* may be a natural alternative to conventional medications for the treatment of type 2 diabetes because of its significant α-amylase and α-glucosidase inhibition (72, 76). Its aqueous extract is very beneficial because it is safe, reasonably priced, and compatible with conventional preparation techniques. Therefore, *T. occidentalis* is a viable option for the development of phytotherapeutics in the treatment of diabetes because of its balanced enzyme inhibition, antioxidant properties, and hormone-modulatory actions (76, 78).

### Acetylcholinesterase (AChE) and monoamine oxidase inhibition activities

*Telfairia occidentalis*'s inhibitory actions against the enzymes acetylcholinesterase (AChE) and monoamine oxidase (MAO) offer encouraging neuroprotective potential, particularly when it comes to treating neurodegenerative diseases like Alzheimer's and Parkinson's (79). With AChE inhibition nearly matching the standard (0.061 ± 0.009 mg/mL) and MAO inhibition marginally less effective than the reference compound (0.155 ± 0.005 mg/mL), the IC50 values of 0.089 ± 0.005 mg/mL for AChE and 0.176 ± 0.003 mg/mL for MAO inhibition indicate moderate potency (80). Because they stop acetylcholine from breaking down and improve cholinergic transmission, AChE inhibitors are essential to Alzheimer's treatment (81, 82). *T. occidentalis* exhibits a favorable AChE inhibitory action in comparison to several therapeutic plants (80). For example, with an IC50 of 0.03 ± 0.08 mg/mL, Chamaecrista mimosoides root extract showed strong AChE inhibition, surpassing *T. occidentalis*; however, it required organic solvents for extraction (80). Similar to *T. occidentalis*, Pistacia falcata had a lower IC50 of 0.22 ± 0.01 mg/mL, demonstrating the latter's higher effectiveness in aqueous formulations (80). It is noteworthy that *T. occidentalis* is well within the effective bracket because numerous medicinal plants from West Africa show AChE inhibition in the range of 0.05–0.25 mg/mL (80). This implies that its phytochemical components, especially alkaloids and flavonoids, might be involved in its neuroprotective properties (83). Because MAO inhibitors preserve neurotransmitters like dopamine and serotonin, they are useful in the treatment of depression and Parkinson's disease (84, 85). *T. occidentalis* has a little weaker MAO inhibitory activity (IC50: 0.176 mg/mL) than the standard (0.155 mg/mL), but it is still within a therapeutically relevant range (80). With almost total inhibition at 2 mg/mL concentrations and an IC50 of 23.37 ± 0.63 μg/mL for antioxidant activity, Pistacia falcata demonstrated higher MAO-B inhibition than MAO-A (80). Although *T. occidentalis* lacks this strength, its aqueous extract provides a more secure and convenient option for conventional use. Other plants, like Buddleja salviifolia and Salvia tiliifolia, have demonstrated MAO inhibition, although they must be extracted with organic solvents to produce this effect (86). This establishes *T. occidentalis* aqueous extract as a viable and efficient choice for Ethnomedicinal uses. With IC50 values that are comparable to those of conventional medications and better than those of several unrefined plant extracts (79, 80, 87), *Telfairia occidentalis* has strong inhibitory action against both the AChE and MAO enzymes.

### *In vivo* study

Scientists are becoming more interested in the possibility of *Telfairia occidentalis* as a treatment for the hormonal and histological abnormalities that monosodium glutamate (MSG) causes in albino rats. Because of its endocrine-disrupting effects, particularly its elevation of testosterone and oestradiol levels, MSG, a common dietary ingredient, has been linked to the formation of uterine leiomyomas (19). On the other hand, *T. occidentalis* exhibits encouraging corrective qualities, as shown by tissue repair and hormone modulation. Rats given MSG showed noticeably higher testosterone levels, which are indicative of endocrine disturbance linked to the pathophysiology of uterine leiomyoma. Impaired ovarian function and aberrant uterine growth are caused by this hyperandrogenic condition. On the other hand, testosterone and oestradiol levels significantly decreased after *T. occidentalis* extract was administered, indicating a rebalancing of the hypothalamic-pituitary-gonadal axis. Since estrogen dominance is known to promote the growth of fibroid cells, the decrease in oestradiol is very significant. These results are corroborated by studies by Sakpa et al. (88) and Ejike and Ezeanyika (89, 90), which demonstrate that *T. occidentalis* seed and leaf extracts can enhance testicular secretory capacity in male rats, raise testosterone-to-estradiol ratios, and alter sex hormones. The plant's diverse phytochemical composition, which includes flavonoids, alkaloids, and saponins, is thought to be responsible for these effects. These compounds include anti-inflammatory and antioxidant qualities that may have an impact on steroidogenesis. The uterus and ovaries in the *T. occidentalis*-treated group showed notable structural improvements in photomicrographs. Secondary, antral, and atretic growing follicles were seen in the ovarian cortex, suggesting that folliculogenesis had resumed. Restored ovarian function and hormonal responsiveness are suggested by the appearance of luteinized cells and mild vascular congestion. The presence of proliferative endometrial cells inside a moderately infiltrated stroma indicates active tissue remodeling, despite the uterus's epithelial covering displaying indications of vacuolation and degeneration. These results are consistent with earlier observations that *T. occidentalis* preserves endometrial integrity and aids in uterine healing.

The anti-leiomyoma and hormone-regulating effects of a number of additional medicinal herbs have been studied (2). The combined activity of *T. occidentalis*—hormonal correction and histological repair—sets it apart from the others. *T. occidentalis* and T. tetraptera both exhibit potent hormonal effects (91), but *T. occidentalis* seems to provide more thorough ovarian and uterine healing. *T. occidentalis*'s capacity to regulate inflammatory and oxidative stress pathways may be a contributing factor to its effectiveness. Tissue damage results from MSG's induction of lipid peroxidation and disturbance of cellular homeostasis (92). Flavonoids and phenolic compounds, two of *T. occidentalis*'s antioxidant ingredients, probably scavenge free radicals and stabilize cell membranes, protecting reproductive tissues in the process (93). Additionally, its impact on gonadotropins, specifically FSH and LH, points to a regulatory role for the hypothalamic-pituitary axis (94, 95). To stop fibroid growth and restore regular ovarian cycles, this is essential. Through hormonal rebalancing and histological restoration, *Telfairia occidentalis* shows great therapeutic promise in reversing MSG-induced uterine leiomyoma. It provides a comprehensive approach to reproductive health, and its effectiveness compares favorably with that of other therapeutic medicinal plants (2, 23, 96, 97). These results underline the need for additional clinical validation and encourage its incorporation into complementary medicines for the management of fibroid disease.

### Phytochemistry

The HPLC analysis reveals a rich profile of bioactive substances, including phenolic acids, flavonoids, carotenoids, and other nutraceuticals. These compounds may have therapeutic use in the treatment of uterine leiomyomas and hormone imbalance. Gallic acid is unique among the phenolic acids found because of its potent antiproliferative and antioxidant qualities. Gallic acid has been demonstrated to cause apoptosis and stop the formation of aberrant cells, which may help prevent the development of fibroid tumors (98). Through their anti-inflammatory and free radical-scavenging properties, caffeine and ferulic acid also help to reduce the oxidative stress associated with fibroid pathology (99). Another phenolic substance found, chlorogenic acid, has shown promise in influencing the metabolism of estrogen, a crucial hormonal route linked to the development of fibroid tumors. Hormone levels can be stabilized and the excessive cellular proliferation associated with uterine leiomyomas can be avoided with this control (100). Beta-carotene, lutein, and zeaxanthin are examples of carotenoids that have strong antioxidant properties that shield reproductive cells from oxidative damage. According to Ross et al. (101), these substances are known to control hormonal signaling and inflammatory mediators, which may improve the uterine environment and lower the risk of fibroid disease. It is encouraging that flavonoids like rutin and kaempferol are present. By reducing the proliferation of fibroid cells and altering the expression of the estrogen receptor, kaempferol has shown anti-fibroid action (102). According to Ganeshpurkar and Saluja (103), rutin improves vascular integrity and may also have an impact on estrogen activity, which helps maintain hormonal balance and improve uterine blood flow.

Beta-sitosterol, a plant sterol with immune-modulatory and hormone-regulating qualities, is another important substance that has been found. It is a useful substance for treating fibroid-related symptoms and hormonal imbalances because of its capacity to lower systemic inflammation and restore normal estrogen levels (104). Furthermore, inositol is essential for the regulation of insulin and estrogen and is frequently used to treat polycystic ovarian syndrome (PCOS), a condition that has characteristics with uterine leiomyomas in terms of hormonal disturbance. Hormonal profiles and ovulatory function have been demonstrated to improve with inositol supplementation (105). These substances also show encouraging anticancer properties via a number of different pathways. By altering cell cycle proteins, gallic acid causes apoptosis and inhibits the growth of cancer cells, particularly breast and prostate cancers (98). Both rutin and kaempferol cause tumor cells to undergo apoptosis and block angiogenesis; kaempferol is selective for malignant cells (102, 103). The disruption of oxidative stress and inflammatory pathways that promote cancer progression by caffeine, ferulic acid, and chlorogenic acid may prevent metastasis and aberrant cell proliferation (99, 100). While beta-sitosterol reduces cancer cell viability through hormone modulation, beta-carotene and other carotenoids scavenge free radicals and may prevent carcinogenic processes (101, 104). These natural bioactive compounds work together to provide a multi-targeted approach to cancer prevention and therapeutic assistance through their immune-modulating, antiproliferative, and antioxidant properties. As a result, the compounds detected by HPLC exhibit a strong combination of hormone-regulating, anti-inflammatory, and antioxidant qualities. In addition to stabilizing hormonal imbalances, their combined efforts may help lower the risk, size, or symptomatic impact of uterine leiomyomas. This offers strong proof of these natural compounds' therapeutic potential for women's reproductive health.

### Molecular docking studies and molecular dynamics simulation of identified compounds against the target

By regulating iron metabolism, inflammation, and oxidative stress, STEAP4 may have an impact on fibroid pathogenesis by encouraging smooth muscle proliferation and extracellular matrix formation, two important aspects of fibroid growth and development (106–108). The molecular docking analysis was conducted to evaluate the binding affinities of the *T. occidentalis* identified compounds against the 6HD1 protein target. The docking scores, which reflect the estimated free binding energy (kcal/mol), revealed that *beta-carotene* exhibited the strongest binding affinity with a docking score of −9.8 kcal/mol, followed closely by *lutein* and *rutin* at −9.7 and 9.5 kcal/mol each. Kaempferol *and* zeaxanthine also demonstrated notable binding potentials with scores of −9.2 kcal/mol. Other bioactive compounds, including *beta-sitosterol* (−8.9 kcal/mol), *catechin* (−8.3 kcal/mol), and *chlorogenic acid* (−8.8 kcal/mol), showed moderate binding affinities. In contrast, lower binding affinities were observed for caffeic acid *and* tryptophan *(both* −*6.7 kcal/mol)*, protocatechuic acid, ferulic acid *(*−*6.6 kcal/mol)*, p-coumaric acid, vanillic acid *(each* −*6.4 kcal/mol), and* sinapic acid (−6.3 kcal/mol). The weakest binding interactions were recorded for *gallic acid* (−6.1 kcal/mol), *4-Hydroxybenzoic Acid*, and *Inositol* (each −6.0 kcal/mol). Overall, the results indicate that carotenoids (Beta-Carotene, Lutein, Zeaxanthine) and flavonoids (*Rutin, Kaempferol*) exhibit superior binding affinity toward the 6HD1 protein, suggesting their potential as promising candidates for further *in vitro* and *in vivo* evaluation. Conversely, phenolic acids and other small molecules displayed comparatively lower binding affinities, indicating a possibly lesser contribution to inhibitory activity against this target. The two top-ranked compounds (Lutein and lutein) from the docking analysis were further subjected to interactive analysis. Beta-carotene was stabilized in the binding to STEAP4 (6HD1) by mainly hydrophobic interactions, including pi-sigma contacts with Phe236 and Trp319, pi-alkyl contacts with Trp319, Phe236, Leu306, while the remaining interactions, including those with Tyr307, Ile240, Ile313, Arg314, Tyr316, Tyr315, Ala239, Val310, and Tyr315 were from alkyl contacts. Lutein was also stabilized by hydrophobic interactions, including pi-sigma contact with Trp319, pi-alkyl contacts with Trp319, Phe236, Leu306, and Tyr307, and alkyl contacts with Ala239, Leu306, Val310, Phe236, Tyr307, Trp319, and Arg314.

Molecular dynamics (MD) simulations are a crucial computational approach for exploring ligand-protein interactions, providing insights into binding mechanisms, stability, and dynamic behavior at the atomic level (109). The RMSD measures the overall structural stability throughout the simulation, where a low or stable RMSD value indicates the formation of a well-structured and stable complex (109). The RSMD systems for both complexes equilibrated around 10 ns, after which both systems presented different progression until the end of the simulation. The lutein-bound complex presented a higher RMSD (8.27 Å), suggesting greater fluctuation or structural drift from the initial conformation during simulation. This could indicate less stability or more flexibility of the complex. Compared to 6HD1_lutein, the beta-carotene-bound system presented a lower RMSD (6.33 Å) with a smaller standard deviation, implying it maintains a more stable and consistent structure throughout the simulation (109). The flexibility of each protein residue is measured by RMSF. Reduced flexibility and a more rigid structure are indicated by lower RMSF values (110). The plot of RMSF systems for both complexes presented the highest fluctuation around amino acid residues between 289 and 337. With the mean RMSF values, the 6HD1_lutein complex had a higher average RMSF of 3.29 ± 1.47 Å, indicating higher residue flexibility, with some residues showing considerable fluctuations, while the beta-carotene complex had a lower RMSF of 2.86 ± 1.24 Å, suggesting lower residue mobility and more restricted atomic motions (111). RoG measures the compactness of the protein-ligand complex. A lower RoG typically indicates a more compact and stable protein structure. A higher RoG may suggest unfolding or structural expansion, while a lower RoG suggests a more stable, compact structure (112). Lutein-bound 6HD1 system shows a higher RoG (35.99 Å), indicating a slightly more expanded or less compact structure, while the beta-carotene-bound 6HD1 system has a lower RoG (34.56 Å), suggesting the complex remains more compact and structurally tighter during simulation. The binding of beta-carotene may have promoted a more compact and organized protein structure, which may relate to better conformational stability (113).

The amount of protein surface exposed to the solvent is measured by SASA. More exposure is indicated by a higher SASA, whereas a more compact or buried building is indicated by a lower SASA (114). The SASA plots for both systems were equilibrated at the beginning of the simulation with a variable fluctuation during the period of the simulation. The lutein-bound system showed slightly higher SASA, meaning more surface area is exposed to solvent, which could be linked to less tight binding or structural loosening during simulation, while the beta-carotene-bound complex exhibited lower SASA, suggesting greater burial of residues and potentially more stable, compact binding. The slightly higher mean SASA value for lutein lutein-bound system suggests greater solvent exposure, potentially indicating an expanded protein structure or greater exposure of hydrophobic residues, while beta-carotene binding results in a more shielded conformation with reduced surface area exposed to solvent (114). Hydrogen bonds refer to the number of hydrogen bonds formed between the protein and the ligand. H-bonds are crucial for ligand-protein stability; a higher mean H-bonds number indicates stronger interactions. The plots for the number of hydrogen bonds in both systems were equilibrated at the beginning of the simulation, with a variable fluctuation during the period of the simulation. The 6HD1_lutein complex formed an average of 223.15 ± 11.3 hydrogen bonds, slightly more than beta-carotene (220.76 ± 10.7). The lutein complex engaged in a higher number of hydrogen bonds, indicating potentially stronger polar interactions with the protein. However, despite more hydrogen bonds, lutein induced greater overall flexibility and expansion, possibly due to weaker hydrophobic packing or induced strain. The beta-carotene-bound system presented slightly fewer hydrogen bonds but contributed to better global stability, as seen in lower RMSD, RMSF, and RoG (115). Overall, beta-carotene encourages a more compact, stable structure with less flexibility and less overall motion, indicating that it might stabilize the protein structure better in the simulated environment. These variations demonstrate that global conformational dynamics must also be considered and that binding affinity (hydrogen bonds) alone does not entirely determine complex stability.

The binding free energy measures the energy difference between the bound and unbound components of a complex (ligand and receptor); the larger the negative value, the stronger the ligand's binding to the protein (116). During the initial phases of drug discovery and development, binding free energy estimates offer thorough details on the binding mechanisms of the top docked compounds (117). The 6HD1_beta carotene (−31.91 ± 5.47) (−29.40 ± 3.83 kcal/mol) presented higher binding free energy when compared to the 6HD1_lutein system, suggesting that it has a more stable and favorable binding interaction with the protein. Both systems exhibited favorable Van der Waals interactions, with 6HD1_beta_carotene having slightly stronger non-polar interaction energy (more negative value), implying better hydrophobic packing within the binding site. Both systems show weak electrostatic contributions, but 6HD1_lutein demonstrates slightly stronger and favorable electrostatic interactions than 6HD1_beta_carotene. Both systems gain stabilization from non-polar solvation, with 6HD1_beta carotene showing a slightly more favorable hydrophobic effect. 6HD1_beta carotene presented better gas-phase binding affinity, driven mainly by stronger van der Waals forces. 6HD1_beta carotene faced a lower solvation penalty, enhancing its binding favorability. Together, the stronger van der Waals interactions, lower solvation penalty, and greater overall stabilization explain System 2's superior binding affinity.

The per-residue energy decomposition analysis indicated that lutein exhibited a highly favorable binding energy of −17.07 kcal/mol, primarily driven by strong van der Waals interactions (−18.22 kcal/mol), despite a slight unfavorable polar solvation effect. Met220 also contributed notable stabilization (−2.12 kcal/mol), mainly through van der Waals forces. Residues such as Thr217, Ala221, and Val292 provided moderate stabilization (−1.24 to −1.59 kcal/mol), with minor electrostatic and balanced solvation contributions. Additional residues, including Tyr209, Tyr289, Ile222, and Leu288, showed small but favorable effects (−1.07 to −1.20 kcal/mol), largely due to hydrophobic contacts. Overall, lutein's binding is predominantly stabilized by van der Waals forces, with minimal electrostatic influence and variable solvation effects. Similarly, beta-carotene demonstrated the most favorable binding energy (−18.98 kcal/mol), also dominated by van der Waals interactions (−18.90 kcal/mol), with minimal electrostatic contributions and moderate polar solvation penalties. Key stabilizing residues included Leu288, Ile293, and Tyr298 (−1.2 to −1.6 kcal/mol), primarily through hydrophobic interactions. Phe284, Ile222, Phe218, and Val299 offered moderate stabilization, while Leu285, Ala221, and Tyr289 contributed minimally (−0.6 to −0.9 kcal/mol). These findings suggest that hydrophobic contacts and van der Waals forces are the principal drivers of ligand-protein complex stability, with beta-carotene playing a central role in enhancing binding affinity.

Overall, beta-carotene and **l**utein have emerged as potential modulators of STEAP4 based on a combination of computational evidence, including high molecular docking scores, favorable stability profiles from molecular dynamics (MD) simulations, and higher favorable binding free energies derived from MM/GBSA analysis. The high docking scores are indicative of effective complementarity between the ligand and protein in terms of shape, hydrophobic contacts, and potential key interactions (118). The stability of the ligand-protein complexes, as validated using molecular dynamics (MD) simulations, revealed that both beta-carotene and lutein formed stable complexes with STEAP4 over the simulation period. Phytochemicals like Beta-Carotene and Lutein, known for their antioxidant and anti-inflammatory properties, align well with the functional modulation of STEAP4. This computational evidence suggests their potential **t**herapeutic relevance in metabolic and inflammatory disorders where STEAP4 is a key player (119–121). This study presents the first comprehensive evidence of *Telfairia occidentalis* exhibiting potent antifibrotic activity in MSG-induced uterine leiomyoma models and synergistic interactions between beta-carotene and lutein, phytoconstituents of *Telfairia occidentalis*, with STEAP4, a fibroid-associated enzyme, highlighting a novel mechanism of action.

### Limitation

This study employed a single treatment group receiving 100 mg/kg of *Telfairia occidentalis* extract, providing initial biological insights but limiting dose-response evaluation. Future studies should investigate multiple doses to establish therapeutic and toxicity thresholds. Additionally, due to the variability of compounds in *Telfairia occidentalis*, the results may not fully represent the detailed mechanistic responses of these compounds, as gene expression changes in women's reproductive organs could involve broader molecular interactions.

## Conclusion

For the first time, *Telfairia occidentalis* shows significant antifibrotic activity in MSG-induced uterine leiomyoma rats. The extract enhanced ovarian morphology, partially repaired uterine integrity, and markedly altered testosterone and oestradiol levels. Chlorogenic acid, gallic acid, kaempferol, beta-sitosterol, rutin, beta-carotene, lutein, zeaxanthin, inositol, tryptophan, and ferulic acid are all detected in the HPLC chromatogram, indicating possible therapeutic benefit against uterine leiomyomas and hormonal imbalance. Strong interactions between beta-carotene and **l**utein, and fibroid-related enzymes (STEAP4) were found by computational docking, indicating a treatment profile that works in synergy. These results provide scientific support for the traditional use of *T. occidentalis* and point to its potential for creating safe and efficient phytotherapeutics for the treatment of fibroid disease.

## Data Availability

The original contributions presented in the study are included in the article/supplementary material, further inquiries can be directed to the corresponding author.
